# Case Report: Gene Heterogeneity in the Recurrent and Metastatic Lesions of a Myxoid Chondrosarcoma Patient With Aggressive Transformation

**DOI:** 10.3389/fgene.2022.791675

**Published:** 2022-07-14

**Authors:** Xuanhong He, Yitian Wang, Chang Zou, Chuanxi Zheng, Yi Luo, Yong Zhou, Chongqi Tu

**Affiliations:** ^1^ Department of Orthopedics, Orthopedics Research Institute, West China Hospital, Sichuan University, Chengdu, China; ^2^ Bone and Joint 3D-Printing & Biomechanical Laboratory, Department of Orthopedics, West China Hospital, Sichuan University, Chengdu, China

**Keywords:** genetic heterogeneity, extraskeletal myxoid chondrosarcoma, NR4A3, treatment, recurrent and metastatic tumor

## Abstract

Extraskeletal myxoid chondrosarcoma (EMC) is a rare soft tissue sarcoma. In view of the indolent course throughout the prolonged natural history of EMC, it was considered as a low-grade soft-tissue sarcoma. However, recent studies have revealed a high recurrence and metastatic potential in EMC, and the invasiveness of EMC may progress during the protracted clinical course. The mechanism for this aggressive transformation remains unknown. Here, we present a rare case of EMC with aggressive behavior. This case was confirmed *via* pathology and NR4A3 fluorescent *in situ* hybridization. To verify the genetic characteristics of this rare case, a total gene sequencing analyses was performed in the recurrent and metastatic lesions. Intriguingly, different gene mutations were determined in the recurrent and metastatic lesions, which implied the genetic heterogeneity among the different lesions might be related to the aggressiveness of EMC. Furthermore, we discuss a few potential agents against the mutated genes in this case, which may provide novel insights regarding the targeted therapy of EMC.

## Introduction

Extraskeletal myxoid chondrosarcoma (EMC) is a rare mesenchymal malignancy with an incidence of 1 case per 10 million (Wilson et al., 2019; Stacchiotti et al., 2020). EMC mostly affects the deep soft tissue of proximal extremities and limb girdles, while rarely in the foot (Wilson et al., 2019; Stacchiotti et al., 2020). This neoplasm is always described as low-grade tumor because it has an indolent course throughout the prolonged natural history. Nevertheless, recent studies revealed that EMC may be a more aggressive malignant tumor neoplasm owing to the high rate of local recurrence and distant metastasis even after radical resection (Meis-Kindblom et al., 1999; Hisaoka and Hashimoto, 2005; Chiusole et al., 2020). For patients with repeated recurrences, the interval between recurrences was gradually shortened, suggesting that the EMC may become more invasive after relapsed (Paioli et al., 2021). Several clinical features have been shown to be associated with the poor prognosis, including age, tumor size, tumor location, high grade, metastasis, and inappropriate initial surgery (Meis-Kindblom et al., 1999; Kawaguchi et al., 2003; Chiusole et al., 2020; Paioli et al., 2021). However, the prognostic significance of these factors vary in different studies and the mechanism of invasive progression of EMC was seldomly discussed.

The translocation of the NR4A3 gene is the characteristic of the EMC, in which the NR4A3 mainly fuses with either EWSR1 or TAF15 (Hisaoka and Hashimoto, 2005). Previous studies have reported that the NR4A3-TAF15 genotype has difference in transcriptional profile and response to drugs from NR4A3-EWSR1, and patients with NR4A3-TAF15 always present a poor prognosis (Agaram et al., 2014; Brenca et al., 2019). Besides, genetic analysis of EMC lesions also contributes to the understand the molecular characteristics of EMC and explored potential therapeutic targets (Davis et al., 2017). Therefore, exploring the genetic profile of EMC patients may help further understand the molecular mechanisms and specific genetic features of EMC, especially of the aggressive transformation of this tumor. Whereas, to the best of our knowledge, none of the published cases had investigated the genetic profile of the patient with multiple recurrent lesions and distant metastases. Here, we present a case which initially demonstrated a typical indolent course of EMC, but rapidly progressed after the marginal resection and eventually died of pulmonary failure. At the same time, in order to better understand the clinical course and genetic characteristics of EMC patient, we also investigated the genetic heterogeneity of different lesions using the next -generation sequencing analysis.

## Case Description and Genetic Analysis

**FIGURE 1:**
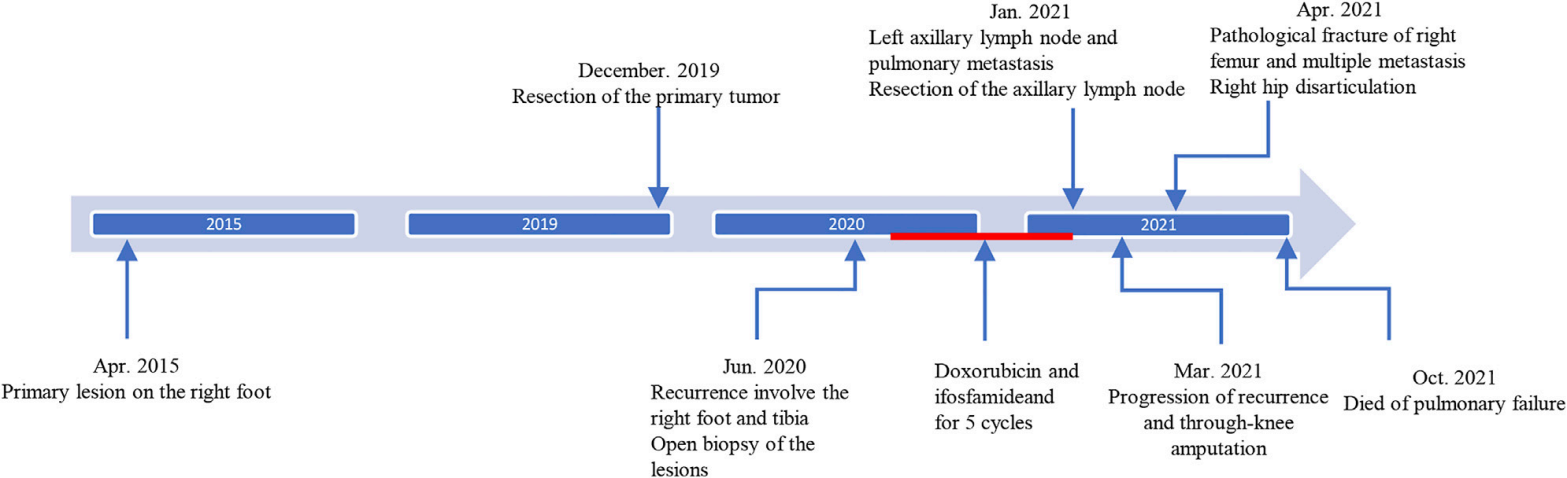
The timeline of the disease.

**FIGURE 2:**
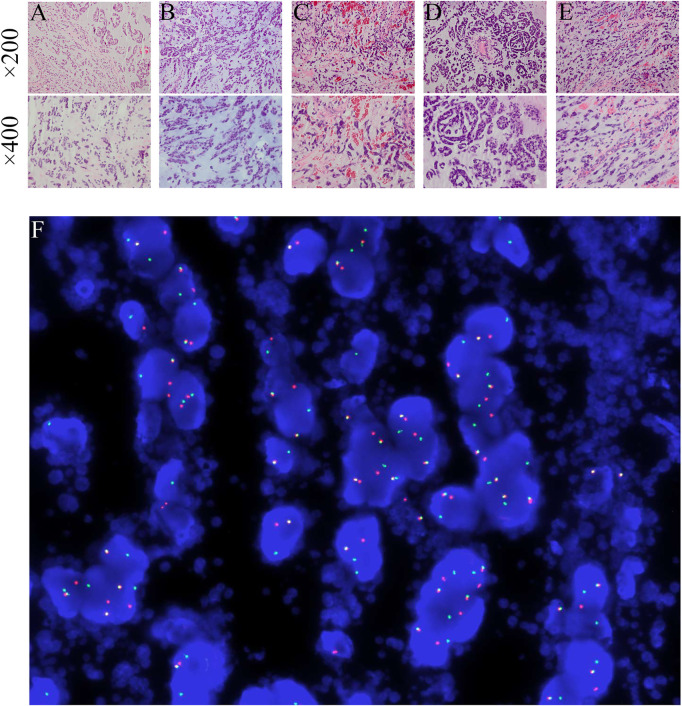
**(A–E)** The pathological findings of the primary and metastatic tumors obtained from five surgeries (HE staining, ×200 magnification and ×400 magnification). The panels exhibit a characteristic cellular round cell tumor with a background of abundant myxoid matrix comprised with numerous lipoblasts. **(F)** NR4A3 rearrangement by break-apart FISH showing signals detected in the form of split signals (centromere, orange; telomere, green), in contrast to normal fused signals. DAPI × 1000.

A 46-year-old male patient complained a painless mass with slow progreesion at the right ankle over 6 years (Figure 1). The mass gradually enlarged to 3.1 cm × 3.2 cm and limited the mobility of the right ankle, then the patient visited local hospital for treatment. The diagnosis of benign soft tissue tumor was considered and a marginial resection of mass was perfomred. However, the pathological exmation revealed the diagnosis of extraskeletal myxoid chondrosarcoma (Figure 2A). The patient was advised regularly follow-up without any further treatment.

**FIGURE 3:**
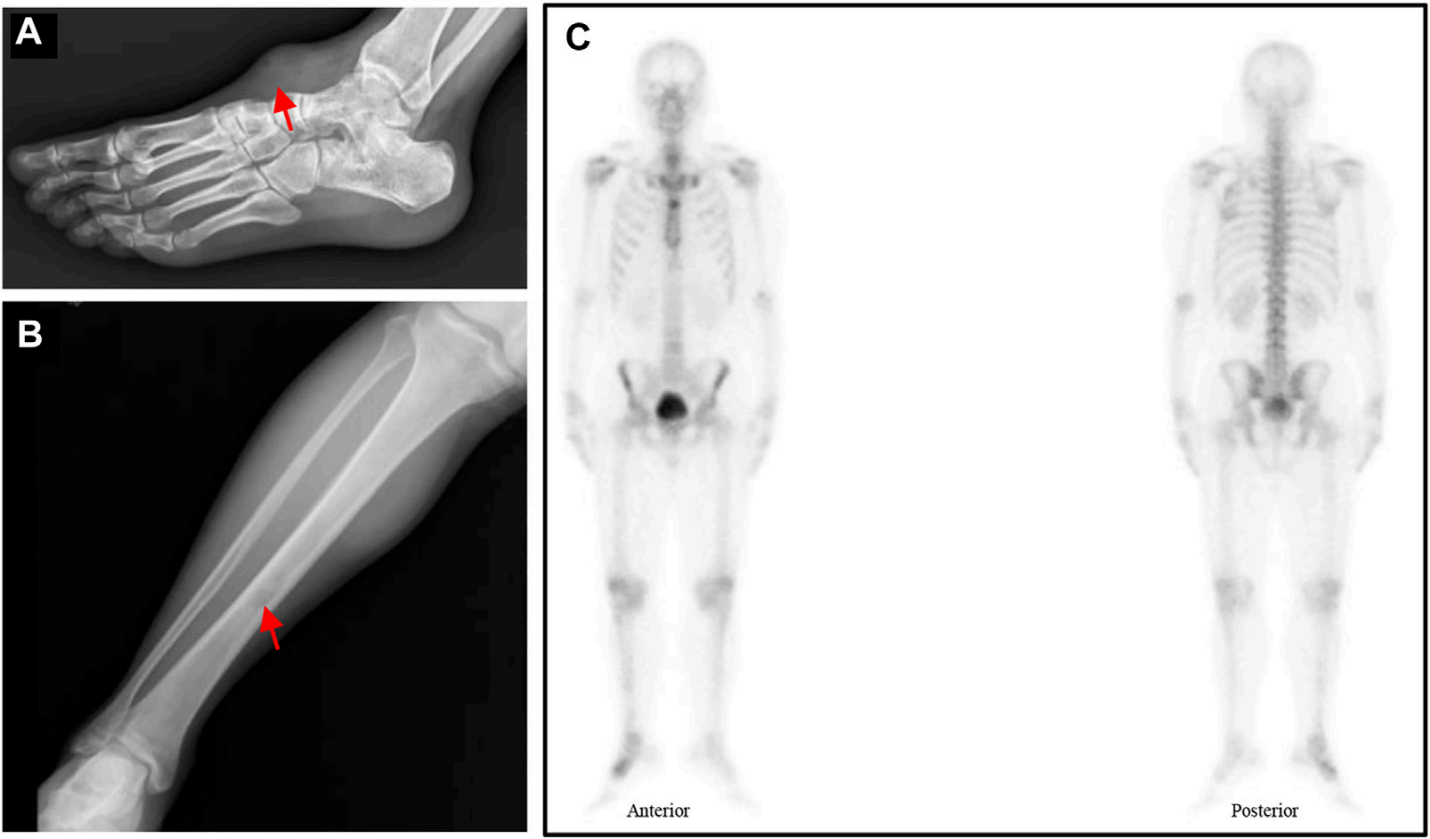
**(A–B)** Radiograph showed a mass on the back of the right foot and a low intensity at the middle of the tibia (red arrow). **(C)** The SPECT displayed a hypermetabolism foci on the right foot.

After 6 months, a soft tissue mass recured at the first surgical site and the patient was referred to our department. Physical examination revealed multiple soft mass on the dorsum of the right foot, with the largest mass of about 3.4 cm × 3.5 cm. The radiograph showed a mass on the back of the right foot, and a low intensity at the middle of the tibia (Figures 3A,B). Chest Computed tomography (CT) showed no lung metastasis, and Single Photon Emission Computed Tomography (SPECT) only presented diffusely increased activity in the right foot without distant metastasis (Figure 3C). Subsequently, a incisional biopsy of the lesion at the right foot and right tibial was performed. Pathological finding showed a characteristic cellular round cell tumor with a background of abundant myxoid matrix comprised with numerous lipoblasts (Figure 2B). The FISH test identified the translocation of NR4A3, that confirmed the diagnosis of extraskeletal myxoid chondrosarcoma (Figure 2F). Nonetheless, the patient refused further surgical resection, and the chemotherapy consist of doxorubicin and ifosfamideand was administrated for five cycles after Multi-Disciplinary Treatment.

**FIGURE 4:**
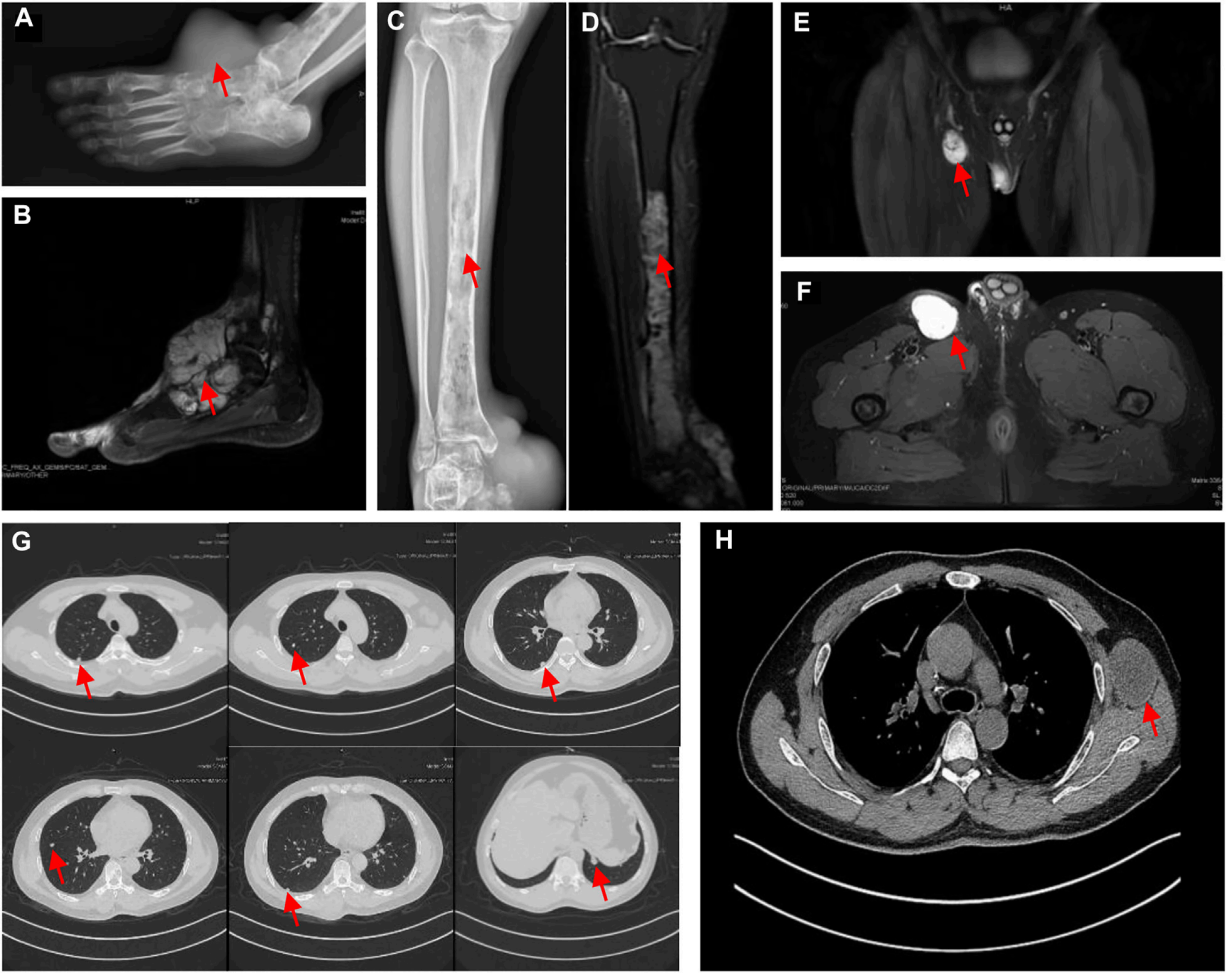
**(A)** The radiograph showed an increase in size compared with Figure 1A and implication of the tarsus and talus; the radiograph revealed that pathological fracture in the middle of the right femur with a heterogeneous low signal (red arrow) in the medullary cavity. **(B)** T2-weighted MRI showed a heterogeneous high homogeneous signal in T2WI with a clear border. **(C)** and **(D)** the X-ray and MRI revealed the metastasis in the tibia and fibula. Consecutive bone destruction was seen in the middle and lower segment of the tibia and the distal fibula. **(E)** and **(F)** The MRI (coronal and transverse sections) displayed the metastasis in right inguinal lymph nodes **(G)** CT scan of the chest showed the pulmonary metastasis. **(H)** CT showed the left axillary lymph node metastasis.

**FIGURE 5:**
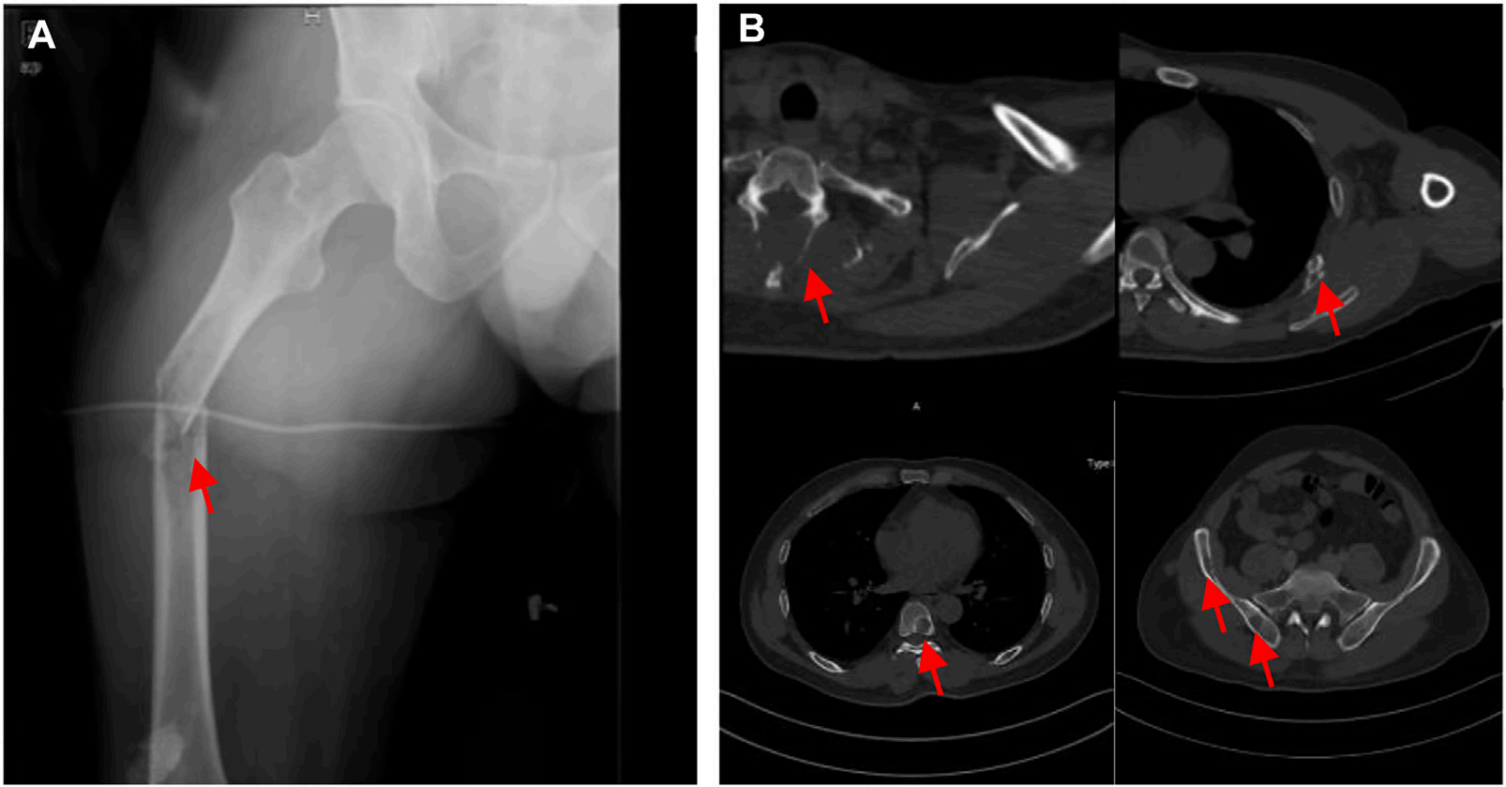
**(A)** Pathological fracture of the right femur. **(B)** Metastases at the ribs, vertebral and ilium on CT.

However, the chemotherapy showed limited efficacy in controlling the disease progression. After five cycles chemotherapy, the local recurrent lesion progressed and involved the right foot, tibia, and fibula with a complete loss of right ankle function (Figures 4A–D). In addition, CT and magnetic resonance imaging (MRI) also revealed multiple suspicious metastases in the right inguinal lymph node (Figures 4E,F), pulmnoary (Figure 4G) and the left axillary lymph nodes (Figure 4H). In order to control the disease progression, the patient was manged with through-knee amputation and right inguinal lymph node excision. The pathological result confirmed the metastase of EMC in inguinal lymph node (Figures 2C,D). Unfortunately, a pathological fracture occured in the femur just 1 month after the through-knee amputation. Radiograph showed a new metastase in the middle shaft of the right femur, while CT showed multiple bone metastases including ribs, vertebral body and ilium (Figures 5A,B). Considering the involvement of vascular and nerves, the patients was treated with right hip disarticulation to control the lesion and improve the quality of life (Figure 2E). Eventually, the patient was reluctant to undergo palliative chemotherapy and died of respiratory failure.

To investigate genetic alterations of this rare case, we performed somatic tumor testing on soft tissue metastasis lesions acquired from the recurrent and metastatic lesions. For the reason that the patient received resection of the primary tumor in local hospital, it`s unavailable to perform NGS testing to the primary lesion. Cell DNA of tumors (frozen in liquid nitrogen and stored at −80°C) were extracted from fresh biopsies using a DNA extraction kit (TIAN-GEN, Beijing, China). In parallel, genomic DNA was extracted from whole peripheral blood referring standard phenol-chloroform extraction procedures to help determine germline variation. Next generation sequencing (NGS) of 999 oncogenes and tumor suppressor genes from the Cancer Group (illumina NovaSeq 6,000 system, United States) was utilized to assess cancer-specific mutation status. Sequencing depth and Q30 of the NGS were at least 3622X and 92.12%, respectively. The coverage and mapping rate were at least 99.92 and 99.97%, respectively. The NGS gene panel was showed in the Supplementary Table S1. Sequencing analysis of the four samples demonstrated that all the lesions were microstatellite-stable tumor with low tumor mutational burden (1.52mut/Mb, <1muts/Mb, <1muts/Mb, 1.92muts/Mb, respectively). Immunohistochemical staining of four samples were negative for PD-L1 expression. Genomic alterations identified in different lesions and possible therapy agents were showed in Table 1. Panel sequencing analysis of the 999 genes identified somatic mutation in three gene with therapeutic implication, including copy number gain in EGFR, copy number gain in FBXW7, exon4, c.585-2A > T, and copy number gain in MET in the recurrences. Importantly, the therapeutic implication of the somatic mutation indicated that the patient might be sensitive to anti-EGFR monoclonal antibody (anti-EGFR-mAb), mTOR inhibitor, and Crizotinibc and Bozitinibc (Table 1). The somatic mutation of MDM4 was variant of uncertain significance (VUS). In addition, the bioinformatic programs, including Sorting Intolerant from Tolerant (SIFT), Polymorphism Pheno-typing v2 (PolyPhen-2) were used to evaluate the possible pathogenicity and the effects on protein function of germline variants. The germline variant of the GLANT12 was predicted as “Tolerated” by SIFT and “Damaging” by PolyPhen-2, and that of the SRC was predicted as “Tolerated” by SIFT and “Tolerated” by PolyPhen-2. However, those two germline mutations in recurrence and metastase were classified as variants of uncertain significance (VUS) in ClinVar (http://www.ncbi.nlm.nih.gov/clinvar).

**TABLE 1:** Genomic alterations identified in different lesions with corresponding targeted agents.

Source	Mutation pattern	Gene	Transcript	cHGVS	pHGVS	Exon/Intron	Mutation abundance	Amplification	Mutation type	SIFT function	Polyphen-2 function	Therapeutic implication
Recurrence (Right foot and tibia, pre-treatment)	Somatic	EGFR	NM_005228	—	—	—	—	3.00	copy number gain	—	—	anti-EGFR-mAb^c^, Nimotuzumab^c^, Lapatinib^d^, and Cetuximab^d^
		FBXW7	NM_033632	c.585–2A > T	—	exon4	7.96%	—	missense	—	—	mTOR inhibitor^d^ and Belinostat^d^
	Germline	GLANT12	—	c122G > A	p. R41H	exon1	—	—	het	Tolerated	Damaging	VUS
Metastases (Axillary lymph nodes)	Germline	SRC	NM_198291.1	c.175G > A	p. A59T	exon4	0.70%		missense	Tolerated	Tolerated	VUS
Recurrence (Right foot and tibia, post-treatment)	Somatic	EGFR	NM_005228.3	—	—	—	—	4.13	copy number gain	—	—	anti-EGFR-mAb^c^, Nimotuzumab^c^, Lapatinib^d^, and Cetuximab^d^
		MET	NM_000245.2	—	—	—	—	2.80	copy number gain	—	—	Crizotinibc^c^, Bozitinibc^c^, Cabozantinibd^d^, Volitinibd^d^, Capmatinibd^d^, and Tepotinib^d^
		MDM4	NM_002393.4	—	—	—	—	3.00	copy number gain	—	—	VUS
Metastases (Femoral)	Somatic	FBXW7	NM_033632.3	c.1637C > A	p.S546*	exon10	20.50%	—	missense	—	—	mTOR inhibitor^d^

^a^FDA-approved therapy

^b^Large scale clinical trial-verified therapy

^c^Approved in other caicinomas

d Preclinical studies or case report support; VUS, variants of uncertain significance.

## Discussion

EMC was a low-grade malignancy primary affecting the limbs and limb girdles while seldomly affecting the foot, with 5-years, 10-years, and 15-years survival rates of 82–90%, 65–70%, and 58–60%, respectively (Meis-Kindblom et al., 1999; Stacchiotti et al., 2020). Despite the indolent behavior and a long clinical course, EMC has a high rate of local recurrences and distant metastasis (Chiusole et al., 2020). Here we report a rare EMC deriving from the foot, which developed aggressive during the clinical course with recurrences and multiple metastases (Figure 1). The present case showed that EMC has the potential for a more invasive transition during its protracted clinical course, which may lead to a dismal prognosis.

Due to the extremely low incidence of EMC, there is limited recognition towards the aggressive transition mechanism of EMC. Although factors such as older age, tumor size (>5 cm), tumor location (non-extremities), high grade (increased cell density and atypia), metastatic status, and adequate initial surgery are associated with poor prognosis in different studies, the prognostic significance of those factors remain controversial (Meis-Kindblom et al., 1999; Chiusole et al., 2020). For example, in a univariate analysis of 42 EMC patients, Kawaguchi et al. revealed that adequate initial surgery was the only factor associated with local recurrence (Kawaguchi et al., 2003). Conversely, in a multicenter study of the Italian Sarcoma Group, the distant metastasis-free survival merely depended on the primary tumor size (Paioli et al., 2021). Therefore, there may exist limitations in evaluating the probability of aggressive transformation of EMC with those clinical features. Recently, next generation sequencing showed its potential in identifying the gene difference between primary and metastatic tumors in EMC (Davis et al., 2017). Next generation sequencing technology is an advanced and available tool, and can help to match individual patients’ genetic abnormalities with molecular targeted therapy, promoting the practice of personalized molecular targeted therapy. Therefore, we intend to explore the possible molecular characteristics of this aggressive transition through genetic analysis of different recurrent and metastatic lesions.

In this current case, the results of genetic analysis revealed that gene heterogeneity existed in different recurrences and or metastases (Table 1). Copy number gain in EGFR was founded in recurrences, and was considered to be associated with poor prognosis and have therapeutic implication. EGFR can be structurally activated by the EGFR amplification, which is associated to cell proliferation, migration and differentiation (Yarden and Shilo, 2007; Sun et al., 2009; Grob et al., 2013; Hirsch et al., 2022). In this case, the recurrences exhibited a progressive behavior and developed distal metastases including pulmonary, lymph nodes and bone metastases in a short stage. Except for the aforementioned clinical factors, we speculate that EGFR amplification may also contribute to the development of metastasis in the present EMC case. In a genetic analysis of 55 small cell carcinoma of primary tumors and brain metastases, Menghong et al. found that EGFR amplification was significantly increased in NSCLC patients with brain metastases (Sun et al., 2009). Synchronously, the frequency of EGFR amplification enlargement in the primary tumor gradually increased with the development of brain metastases (Sun et al., 2009). Those findings suggest that EGFR amplification has an important driven effect on the occurrence of metastases in patients with cancers. Similarly, the copy number gain in EGFR was also founded elevated in the recurrence after the chemotherapy, suggesting that this amplification in EGFR may be one of the contributors to the rapid metastasis. However, no-EGFR mutation was found in other metastatic sites. Consistent with previous study, there exists the heterogeneity in the EGFR coincidence rate between different lesions (Massarelli et al., 2007; Sun et al., 2009). Moreover, this heterogeneity may explain the distinction on different treatment responses, and may affect the assessment of anti-EGFR therapy with the mutation of EGFR. Therefore, considering the heterogeneity of EGFR variant in the recurrent and metastatic lesions, the EGFR-mAb therapy may have limited effect in this patient.

Simultaneously, the other significant variant, the copy number gain of MET, was also found in the recurrence. MET amplification is often associated with a propensity to metastasize and poor prognosis in cancer patients, including gastric, esophageal, lung, and head and neck cancers (Lal et al., 2009; Organ and Tsao, 2011; Blumenschein et al., 2012; Kawakami et al., 2014). Additionally, there exist complex relationship between MET and EGFR, which may influence the therapeutic effect of targeted-EGFR drugs. In a study investigating genomic MET status and protein expression in 266 salivary gland carcinomas, Tobias et al. revealed that the copy number gain of MET was positively correlated with copy number gain of EGFR, suggesting that EGFR activation could affect the MET stability (Ach et al., 2013). Interestingly, this effect also seems to be reflected in the therapeutic effect of EGFR inhibitors. Previous studies have shown that EGFR tyrosine kinase inhibitors have limited efficacy in lung cancer patients with high MET levels (Engelman et al., 2007; Cappuzzo et al., 2009; Benedettini et al., 2010; Wu and Shih, 2018). The probably reason may be that MET amplification stimulates the PI3K-AKTmTOR pathway by activating HER3, leading to the tumor growth and invasion independent from the inhibition of EGFR (Engelman et al., 2007). In contrast, additional inhibition of MET may increase the sensitivity to EGFR TKIs, implying that the function of MET also modulates the therapeutic effect of EGFR TKIs (Arteaga, 2007; Engelman et al., 2007). Furthermore, EGFR signaling also appears to be associated with acquired MET kinase inhibitor resistance (Danilkovitch-Miagkova and Zbar, 2002; Cepero et al., 2010). Therefore, for patients with both the mutation of copy number gain in EGFR and MET, the combination of MET and EGFR inhibitors may be beneficial. In this present case, we revealed both EGFR and MET amplification mutations in recurrence, which may co-contribute to the aggressive transformation in this case. Meanwhile, the combination of MET and EGFR inhibitors may benefit this patient.

In addition, missense mutation in FBXW7 was found in the recurrence and femur metastasis. The progression of the recurrence was significantly different from that of the primary lesion which developed slowly. Meanwhile, this patient rapidly developed systemic metastases in a short period of time, in which the femur metastasis even progressed with pathological fracture. Although there are limited studies of FBXW7 in EMC, we found that FBXW7 was correlated with the recurrence and metastasis of several cancers, and even the poor prognosis (Li et al., 2018; Liu et al., 2018). FBXW7 is well-known as a tumor suppressor involved in protein degradation, and inactivation of FBXW7 could lead to abnormal accumulation of oncoproteins, inducing malignant transformation and tumor metastasis (Mao et al., 2008; Welcker and Clurman, 2008; Kar et al., 2021). For example, according to Krittiya et al., the FBXW7 missense mutation was associated with poor prognosis in metastatic colorectal cancer (Korphaisarn et al., 2017). Therefore, we hypothesize that missense mutations in FBXW7 also play a role in the metastatic development in this patient. More important, the mutated FBXW7 could be a potential therapeutic target. As reported, mTOR inhibitor therapy may be suitable for the treatment of patient with FBXW7 inactivation. Interestingly, in several studies about the EGFR monoclonal antibody treatment response in colorectal cancer, FBXW7 mutation was significantly associated with negative treatment response with EGFR monoclonal antibody, indicating that the therapeutic effect of EGFR monoclonal antibody was extremely limited in patients with FBXW7 mutation (Lupini et al., 2015; Rachiglio et al., 2019; Mendelaar et al., 2021).

In this present case, we report a rare EMC primary involving the foot, and developing recurrence and metastasis in a short stage. Through the genetic analysis of recurrent and metastatic lesions, we found three significant genetic mutations which may be associated with this aggressive alteration. In addition, our results also revealed the rare variants in the recurrent and metastatic lesions, such as germline heterozygous mutation in GLANT12 and SRC, and somatic mutation in MDM4. The clinical significance of those variants needs to be further verified. In addition, we also review the histological features of different lesions (including the primary tumor), which revealed that the histological features of the primary tumor, recurrence, and metastasis had no significantly difference (Figures 3A–E). This historical result implies that the heterogeneity mainly exists in the gene level while seldom in the histological level. Therefore, genetic heterogeneity may act as a supplementary prognostic predictor to other clinical characteristics, indicating the need for genetic analysis of EMC patients with recurrence or metastases.

We must acknowledge that our study has some limitations. First, patients are treated with doxorubicin and ifosfamide after relapse, and NGS test results may be interfered by DNA damage caused by chemotherapeutic agents. However, anthracycline-based chemotherapy is the standard front-line regimen for patients with progressing metastatic disease, so it is difficult to exclude the interference of chemotherapy with NGS testing. In contrast, our results reflect the genetic heterogeneity of different lesions in patients treated with chemotherapy, which is more consistent with clinical practice. Second, genetic analysis of the primary tumor could not be performed due to the primary lesion is unavailable for NGS. Whereas, we performed genetic analysis of recurrences or metastases acquired from each resection, revealing that there is genetic heterogeneity between recurrent and metastatic lesions, and this heterogeneity may be responsible for such progressive behavior in this patient.

## Conclusion

EMC is a low-grade malignancy with indolent behavior, while transformation of invasiveness is still likely to occur during its long-term clinical course. Our results reveal that there is genetic heterogeneity in recurrent and metastatic lesions, in which copy number gain of EGFR, copy number gain of MET, and FBXW7 missense mutation may be responsible for this aggressive transition in this case. This case may help to elucidate the molecular mechanism of EMC tumor development and explore the potential therapeutic targets.

## Data Availability

The datasets for this article are not publicly available due to concerns regarding participant/patient anonymity. Requests to access the datasets should be directed to the corresponding author.
