# Supplementary material for: Case Report: Gene Heterogeneity in the Recurrent and Metastatic Lesions of a Myxoid Chondrosarcoma Patient With Aggressive Transformation
Source: Front Genet. 2022 Jul 14;13:791675. doi: 10.3389/fgene.2022.791675 (PMC9330136; doi:10.3389/fgene.2022.791675)
Supplement: Supplementary file 2 [file Table1.DOCX]

Table 1 Genomic alterations identified in different lesions with corresponding targeted agents.

| Source | Mutation pattern | Gene | Transcript | cHGVS | pHGVS | Exon/Intron | Mutation abundance | Amplification | Mutation type | SIFT function | Polyphen-2  function | Therapeutic  implication |
| --- | --- | --- | --- | --- | --- | --- | --- | --- | --- | --- | --- | --- |
| Recurrence  (Right foot and tibia, pre-treatment) | Somatic | EGFR | NM_005228 | - | - | - | - | 3.00 | copy number gain | - | - | anti-EGFR-mAb^c^, Nimotuzumab^c^, Lapatinib^d^, and Cetuximab^d^ |
|  |  | FBXW7 | NM_033632 | c.585-2A>T | - | exon4 | 7.96% | - | missense | - | - | mTOR inhibitor^d^ and Belinostat^d^ |
|  | Germline | GLANT12 | - | c122G>A | p. R41H | exon1 | - | - | het | Tolerated | Damaging | VUS |
| Metastases (Axillary lymph nodes) | Germline | SRC | NM_198291.1 | c.175G>A | p. A59T | exon4 | 0.70% |  | missense | Tolerated | Tolerated | VUS |
| Recurrence (Right foot and tibia, post-treatment) | Somatic | EGFR | NM_005228.3 | - | - | - | - | 4.13 | copy number gain | - | - | anti-EGFR-mAb^c^, Nimotuzumab^c^, Lapatinib^d^, and Cetuximab^d^ |
|  |  | MET | NM_000245.2 | - | - | - | - | 2.80 | copy number gain | - | - | Crizotinibc^c^, Bozitinibc^c^, Cabozantinibd^d^, Volitinibd^d^, Capmatinibd^d^, and Tepotinib^d^ |
|  |  | MDM4 | NM_002393.4 | - | - | - | - | 3.00 | copy number gain | - | - | VUS |
| Metastases (Femoral) | Somatic | FBXW7 | NM_033632.3 | c.1637C>A | p.S546* | exon10 | 20.50% | - | missense | - | - | mTOR inhibitor^d^ |

^a^FDA-approved therapy, ^b^Large scale clinical trial-verified therapy, ^c^Approved in other caicinomas, ^d^Preclinical studies or case report support; VUS, variants of uncertain significance.
